# Impact of Aspirin Intake on Postoperative Survival after Primary Pancreatic Resection of Pancreatic Ductal Adenocarcinoma—A Single-Center Evaluation

**DOI:** 10.3390/biomedicines11051466

**Published:** 2023-05-17

**Authors:** Danilo Hackner, Mirianna Hobbs, Susanne Merkel, Christian Krautz, Georg F. Weber, Robert Grützmann, Maximilian Brunner

**Affiliations:** Department of General and Visceral Surgery, Friedrich-Alexander-Universität (FAU) Erlangen-Nürnberg, 91054 Erlangen, Germany; mirianna_05@hotmail.co.uk (M.H.); susanne.merkel@uk-erlangen.de (S.M.); christian.krautz@uk-erlangen.de (C.K.); georg.weber@uk-erlangen.de (G.F.W.); robert.gruetzmann@uk-erlangen.de (R.G.); maximilian.brunner@uk-erlangen.de (M.B.)

**Keywords:** pancreatic ductal adenocarcinoma, pancreatic resection, overall survival, disease-free survival, aspirin intake

## Abstract

(1) Background: The intake of aspirin (ASS) has been demonstrated to have a relevant impact on the pathogenesis, incidence and outcome in different solid gastrointestinal tumors. However, data on the effect of ASS on the short-term outcome and the long-term survival in patients with pancreatic carcinoma are still limited. (2) Methods: A total of 213 patients who underwent primary resection of PDAC at the University Hospital of Erlangen from January 2000 to December 2018 were included in this retrospective single-center study in total. Patients were stratified according to the aspirin intake into three groups: continuous aspirin intake (cASS), perioperatively interrupted aspirin intake (iASS) and no aspirin intake (no ASS) at the timepoint of surgery. The postoperative outcome as well as long-term survival were compared between the groups. (3) Results: There were no differences regarding postoperative morbidity (iASS: 54% vs. cASS: 53% vs. no ASS: 64%, *p* = 0.448) and in-hospital mortality (iASS: 4% vs. cASS: 10% vs. no ASS: 3%, *p* = 0.198) between the groups. The overall survival (OS) and disease-free survival (DFS) did not differ in the groups when comparing the ASS-intake status (OS: iASS 17.8 months vs. cASS 19.6 months vs. no ASS 21.6 months, *p* = 0.489; DFS: iASS 14.0 months vs. cASS 18.3 months vs. no ASS 14.7 months, *p* = 0.957). Multivariate analysis revealed that age (hazard ratio (HR) 2.2, *p* < 0.001), lymph node-positive status (HR 2.0, *p* < 0.001), R status 1 or 2 (HR 2.8, *p* < 0.001) and differentiation with a grading of 3 (HR 1.7, *p* = 0.005) were significant independent prognostic factors regarding the OS. Moreover, age (HR 1.5, *p* = 0.040), lymph node-positive status (HR 1.8, *p* = 0.002) and high-grade (G3) carcinomas (HR 1.5, *p* = 0.037) could be identified as independent prognostic parameters for DFS. (4) Conclusions: In patients undergoing primary surgery for curative resection of pancreatic carcinoma, the perioperative intake of ASS had no significant impact on postoperative outcome, overall and disease-free survival.

## 1. Introduction

With 4.6% of all cancer mortalities, pancreatic cancer (PC) is the third most common cause of cancer-related deaths worldwide [1,2]. Even if diagnosed in localized stages, the 5-year relative survival is at 42%, amongst the lowest of all cancers [2]. One of the reasons for the persistently impaired prognosis is the aggressive nature of pancreatic carcinoma, which often leads to rapid metastasis even in patients who underwent a primary resection of the pancreatic tumor [3,4].

The mechanism of metastasis formation represents a highly complex interaction of a wide variety of factors. In the context of metastasis, a relevant role is attributed to thrombocytes, since thrombocytosis has often been described as being associated with poorer survival [5,6]. Accordingly, various anticarcinogenic effects could be demonstrated for aspirin as a platelet inhibitor. In addition to the reduction in metastasis formation [7], positive effects of aspirin with regard to a reduction in tumor incidence and an improvement in survival have also been described for a wide variety of entities such as colorectal, gastric and esophageal cancer [8,9,10]. In addition to the inhibition of platelets, these effects are also explained by an inhibition of COX-2, which also is increasingly expressed in PC compared to normal pancreatic tissue [11,12].

To date, the available epidemiological studies present inconsistent results regarding a positive effect of aspirin intake and PC development. Two meta-analyses were able to show that there might be reduced incidence of pancreatic cancer in patients with permanent aspirin use, but no influence was seen on mortality [13,14]. However, it remains unclear whether aspirin intake also has a positive effect in patients with already diagnosed PC considering the overall (OS) and disease-free survival (DFS). Especially patients that underwent surgical resection are susceptible to metastasis formation. Due to the necessary mobilization and manipulation of the tumor, more circulating tumor cells (CTCs) are released into the bloodstream, and the mechanisms of the cell-mediated immunity are weakened by postoperative immunosuppression [15]. The data on the effect of aspirin in this special cohort of patients undergoing primary resection are limited.

The primary aim of this study was to evaluate the impact of low-dose aspirin intake on postoperative outcome as well as the OS and DFS in patients undergoing curative pancreatic resection for PC.

## 2. Materials and Methods

The study included 213 adult patients who underwent primary resection of pancreatic ductal adenocarcinoma (PDAC) at the University Hospital of Erlangen, Germany between 1 January 2000 and 31 December 2018. The interdisciplinary tumor board evaluated all patient cases and determined the pancreatic malignancy to be primary resectable based on the available diagnostics. Patients who received neoadjuvant chemo- or radiotherapy were excluded from the study.

Clinical data of patients were obtained from the clinical information system, while pathological and survival data were retrieved from the Erlangen Cancer Registry of the Department of Surgery. The detailed documentation allowed to classify all specimens according to the eighth edition of the UICC/AJCC TNM staging system [16]. Morbidity was assessed using the Clavien–Dindo classification [17], with major morbidity defined as Clavien–Dindo III, IV, and V. The definitions of the International Study Group of Pancreatic Surgery (ISGPS) were used to define the postoperative pancreatic fistula (POPF), delayed gastric emptying (DGE), and postpancreatectomy hemorrhage (PPH) [18,19,20]. The patients were followed-up for a mean time of 33.6 months (range 0–198 months).

This retrospective study was approved by the local ethics committee (22-165-Br).

### 2.1. Study Design

We analyzed all patients in regard to intake of aspirin on a daily basis at admission. In total, 43 patients took 100 mg of aspirin daily for at least one month before surgery due to mostly underlying cardiac diseases. We further stratified the patients into a group with interrupted (iASS, n = 24) and continuous ASS intake (cASS, n = 19). Interrupted intake was found mainly in the years up to 2010 as it was common practice in our department until then to pause antiplatelet agents preoperatively due to the potential risk of bleeding.

The different groups were compared according to the ASS intake status on OS and DFS. Furthermore, potential outcome parameters were stratified to ASS intake, and prognostic factors of patients for OS and DFS were identified.

### 2.2. Surgical Procedures

All surgical procedures were performed by experienced visceral surgeons with extensive expertise in pancreatic surgery. The surgical approach varied depending on the tumor location. Pancreatic head resection was performed either as pancreatoduodenectomy with resection of the distal stomach (Whipple procedure) or as pylorus-preserving pancreaticoduodenectomy (PPPD) based on the extent of the tumor and the surgeon’s individual decision. Inter-aortocaval lymph node dissection was performed during pancreatic head resection, and inter-aortocaval lymph nodes were evaluated as pM1 in cases of tumor involvement. Resection of the primary tumor was not performed in the presence of intraoperative evidence of liver metastases or peritoneal carcinosis. Splenectomy was performed during distal pancreatectomy, and total pancreatectomy was necessary in a few cases. Additional vascular and multivisceral resections were performed if necessary to achieve an R0 situation. Arterial vascular resection was only performed in exceptional cases. An intraoperative pathological examination of the pancreatic margin was performed in all cases. In case of intraoperatively assessed incomplete resection (R1) and the possibility of achieving an R0 situation, further resection was performed.

### 2.3. Adjuvant Chemotherapy and Follow-Up

In an R0 situation, postoperative chemotherapy increased over the years, recommended to all patients since 2007, Prior to this, it was an individual decision of the interdisciplinary tumor board. In cases of incomplete tumor resection, palliative chemotherapy was offered to all patients. However, some patients declined it, or their general postoperative condition was too impaired to perform chemotherapy. Furthermore, a patient’s condition was pivotal regarding whether chemotherapy was performed either with gemcitabine or 5-FU. If curative resection could be achieved, regular follow-up with CT scans of the thorax and abdomen was advised, quarterly until the 3rd year and thereupon once half a year.

### 2.4. Statistical Analysis

For data analysis, the SPSS software (SPSS, Inc., Chicago, IL, USA) was used. The Student t-test or Mann–Whitney U test were used to compare metric and ordinal data. The Chi-square test was used for categorical data. Overall survival (OS) and disease-free survival (DFS) were calculated for the period between the date of surgery and the date of death or last follow-up, respectively, the period between date of surgery and date of death, the date of local or distant recurrence, or last follow-up. For the calculation of the DFS, only patients with an R0 resection were included (n = 186). Possible factors related to the patients’ OS and DFS were tested using univariate and multivariate analyses. Variables with a *p* ≤ 0.05 in univariate analysis as well as aspirin intake were used for multivariate analysis with a Cox regression model. The Kaplan–Meier method was used for plotting survival curves and compared with the log-rank test. A *p* value of ≤ 0.05 was considered statistically significant.

## 3. Results

### 3.1. Patient Characteristics

A total of 213 patients (median age: 68 years (range 45–89 years), 47% female) were included in our analysis. Preoperative patient characteristics are shown in Table 1. Most of the included patients had no history of ASS intake (n = 170, 80%), while 24 patients were taking ASS with perioperative interruption (11%) and 19 patients had continuous ASS intake perioperatively (9%). Age differed significantly in the no ASS vs. ASS intake groups in general and no ASS vs. iASS vs. cASS intake groups (*p* = 0.013 vs. *p* = 0.039). Moreover, patients with ASS intake suffered from hypertension significantly more often (iASS 88% vs. cASS 79% vs. no ASS 48%, *p* < 0.001) and showed cardiovascular (iASS 33% vs. cASS 42% vs. no ASS 6%, *p* < 0.001) and cerebral comorbidities more often (iASS 17% vs. cASS 11% vs. no ASS 4%, *p* = 0.025). ASA score, BMI, values of alcohol and nicotine abuse, biliary stenting and preoperative lab works—including tumor markers—did not differ between the three groups.

### 3.2. Surgical and Histopathological Details

In terms of surgical procedures, the majority of patients underwent a pancreatic head resection (76%), while 21% had a distal pancreatectomy and 3% had a total pancreatectomy. A total of 28% of patients required additional vascular resection, while 18% needed multivisceral resection. The majority of patients (87%) achieved an R0 resection. The surgical and histopathological data, as well as the TNM stage and R status, were comparable across all three groups (Table 2).

### 3.3. Short-Term Postoperative Outcome Parameters

The postoperative outcome parameters can be found in Table 3. Regarding in-hospital morbidity including POPF, DGE, PPH and length of postoperative stay as well as mortality, there was no significant difference between the three groups. In addition, slightly more than half of the patients in each group received adjuvant chemotherapy (iASS 58%/cASS 53%/no ASS 54%, *p* = 0.907).

### 3.4. Overall and Disease-Free Survival

**Figure 1:**
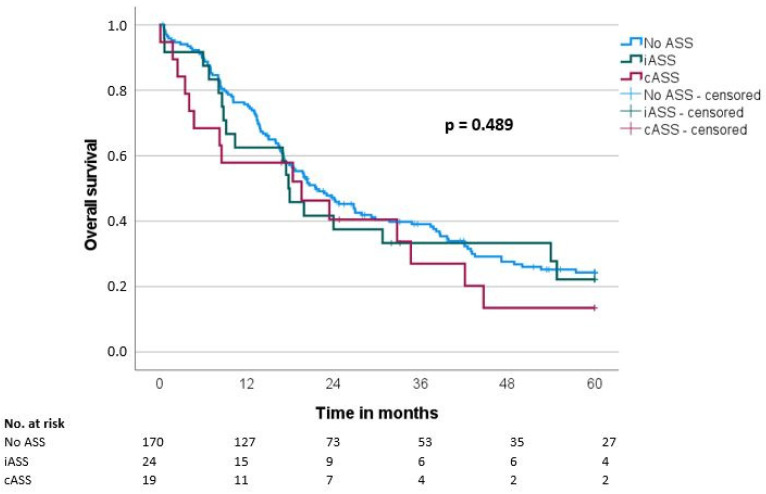
Overall survival (OS) according to the ASS intake (No ASS vs. Interrupted ASS (iASS) vs. Continuous ASS (cASS)) (n = 213).

**Figure 2:**
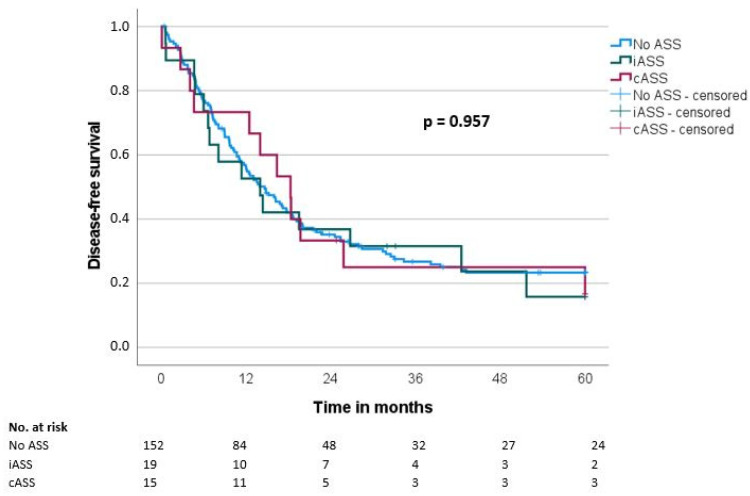
Disease-free survival (DFS) according to the ASS intake (No ASS vs. Interrupted ASS (iASS) vs. Continuous ASS (cASS)) (n = 186 *); * only patients after R0 resection.

Median overall survival (OS) and disease-free survival (DFS) were 20.7 ± 1.9 and 14.7 ± 1.6 months, respectively. There was no significant difference regarding OS and DFS in the groups when comparing the ASS intake status (OS: iASS 17.8 months/cASS 19.6 months/no ASS 21.6 months, *p* = 0.489; DFS: iASS 14 months/cASS 18.3 months/no ASS 14.7 months, *p* = 0.957) (Table 3, Figure 1 and Figure 2).

### 3.5. Prognostic Factors for Overall and Disease-Free Survival

For patients with resected pancreatic carcinoma, the potentially prognostic factors regarding the OS and the DFS are presented in Table 4 and Table 5. Multivariate analysis revealed that age (hazard ratio (HR) 2.2, *p* < 0.001), lymph node-positive status (HR 2.0, *p* < 0.001), R status 1 or 2 (HR 2.8, *p* < 0.001) and high-grade differentiation (G3) (HR 1.7, *p* = 0.005) were significant independent prognostic factors regarding the OS (Table 4). Regarding the DFS, significant independent prognostic factors were age (HR 1.5, *p* = 0.040), lymph node-positive status (HR 1.8, *p* = 0.002) and high-grade carcinomas (G3) (HR 1.5, *p* = 0.037) (Table 5).

## 4. Discussion

In our single-center study, we examined 213 patients with primary pancreatic resection for PDAC with continuous, interrupted or without perioperative ASS intake. The results did not show a significant difference in short- and long-term outcome parameters.

Regarding the short-term outcome, there was no difference in in-hospital morbidity (*p* = 0.448) and mortality (*p* = 0.198). There was no significant higher blood loss in the group of patients with ASS intake compared to patients without it (cASS 750 mL vs. iASS 500 mL vs. no ASS 550 mL, *p* = 0.357). Furthermore, no difference was seen regarding PPH (cASS 0% vs. iASS 0% vs. no ASS 1%) or the re-operation rate (cASS 0% vs. iASS 8% vs. no ASS 10%, *p* = 0.348). These findings are in accordance with several studies, which have reported that, especially in high-risk patients for cardiovascular events, the continuation of low-dose aspirin intake did not increase the risk for bleeding but may have had an impact on reducing adverse cardiovascular events such as myocardial infarction, thromboembolism, cardiac arrest or cardiovascular death [21,22,23,24]. However, other authors reported an increased risk of bleeding and PPH in patients with perioperative ASS intake, not only in patients after pancreatic surgery but also in those that underwent a different noncardiac surgery [25,26]. Fujikawa and Naito performed a systematic review of the literature especially focusing on antithrombotic therapy (ATT) in pancreatic surgery [27]. A total of 37,863 patients who underwent pancreatic surgery and receiving different types of ATT could be included, resulting in a non-increased occurrence of PPH. Focusing especially on perioperative aspirin intake, the authors stated that a continuation should be considered. Gerstein et al. focused on aspirin withdrawal syndrome in perioperative period for patients with an established cardiovascular disease [28]. They reported that the effects of aspirin withdrawal such as the platelet rebound phenomenon and the prothrombotic stage are even increased in the perioperative period, whereas the risk of bleeding while continuing the aspirin intake is minimal in comparison to possible thromboembolic complications. Despite a few high-risk surgical procedures, the authors therefore recommended the continuation of aspirin intake, especially for patients taking it for secondary cardiovascular prevention.

In recent years, increasing evidence has been presented that anti-platelet substances—especially low-dose aspirin—may have a positive effect on different solid tumor entities. Preclinical as well as clinical research was able to show not only the underlying biochemical mechanisms, but also the possible clinical implementations. Zhang et al. concluded that aspirin acts as chemopreventive agent and is moreover able to suppress bioactivities of cancer cells; it thus “is a master regulator of the hallmarks of cancer” [29]. Effects include the inhibition of COX-2, which is responsible for cancer-related inflammation and the supporting prostaglandins, mainly PGE2, and directly interfering with the NF-кB pathway. This might be especially interesting in PC, as it could be shown that a binding of NF-кB to the DNA might be prevented in PC cell lines due to induction of ANXA1, which increases apoptosis and harms proliferation of these cancer cells [30]. Additionally, the anti-platelet effect could have an immediate impact on CTCs and thus distant metastasis. The underlying assumption is that CTCs might be protected by activated platelets [31,32]. Commonly, CTCs are degraded by sheer stress or immune cells, such as natural killer cells, as well as other immune mechanisms [33]. In particular, the immune-mediated degradation might be malfunctioning in this manner.

Promising early data in mainly observational studies regarding the reduced incidence of different gastrointestinal tumors in persons with regular aspirin intake in general could be confirmed in later randomized controlled trials and can also have a positive impact on mortality [34]. However, there is limited data on the effect of ASS on the long-term survival in patients with pancreatic carcinoma. Our analysis of pancreatic carcinoma patients who underwent primary tumor resection could not show a survival advantage for patients taking aspirin compared to those not taking aspirin. Our study is the first to stratify the interrupted and continuous intake of ASS. However, there was no difference between these two groups regarding survival parameters.

To our knowledge, only two other studies with similar patient cohorts are available [32,35]. Pretzsch et al. performed a propensity score matched analysis on 18 patients who received curative intent surgery for PC. They found aspirin to be associated with improved mean OS (46.5 vs. 24.6 months, *p* = 0.006), median DFS (26 vs. 10.5 months, *p* = 0.001) as well as mean hematogenous metastasis-free survival (41.9 vs. 16.3 months, *p* = 0.005) [32]. Comparable results were demonstrated by Tamburrino et al. in a retrospective analysis of 430 patients who underwent pancreatic resection and used chemopreventive agents (i.e., aspirin, statins and metformin) regularly for at least 6 months prior to diagnosis and continued intake after surgery. A total of 69 of these patients took aspirin, which was associated with better DFS (HR: 0.62; *p* = 0.038) [35]. However, as also stated by the authors, the explicit effect of aspirin on the presented results is debatable.

There are several potential factors influencing the association of ASS intake and survival. Generally, it must be assumed that patients taking aspirin due to an underlying disease may face an impaired survival rate in comparison to otherwise healthy individuals, which could obscure a positive survival effect of ASS. The corresponding concomitant diseases such as hypertension, cardiovascular and cerebrovascular comorbidities were also more common in our patient cohort with regular aspirin intake. Furthermore, in our cohort patients taking aspirin were found to be older than patients without ASS intake (cASS: 74 years vs. iASS: 70 years vs. no ASS: 67 years, *p* = 0.039) and age was revealed as independent prognostic survival factor in our analysis and the previous studies [36]. However, a subgroup analysis of patients stratified by age (≤70 years and >70 years) showed again no significant impact of aspirin intake on overall and disease-free survival (≤70 years: OS: *p* = 0.143, DFS: *p* = 0.737; >70 years: OS: *p* = 0.848, DFS: *p* = 0.711). Moreover, imbalances in prognostic factors such as preoperative performance status and pathological stage can explain the different outcomes. However, the ASA score and TNM stages did not differ between the three ASS groups. Furthermore, the postoperative chemotherapy may influence the outcome, as studies show that an adjuvant chemotherapy is associated with prolonged survival [37]. In our cohort, there was no difference in the proportion of adjuvant chemotherapy in the different cohorts (*p* = 0.907). In addition to whether chemotherapy was administered, the type of chemotherapy also plays a crucial role, especially since a relevant improvement in prognosis with FOLFIRINOX was reported [38,39]. Unfortunately, these data are missing from our analysis, which is a relevant weakness of this work. In addition, other relevant factors affecting OS and DFS have been reported, which were not included in our analysis [40]. All the aforenamed reasons could be the cause for a possible effect of ASS being masked in our analysis.

It is important to acknowledge the limitations of our study. Firstly, the data presented are retrospective and were collected from a single center over an 18-year period, which could introduce biases. Additionally, the number of patients included in our analysis is limited. Secondly, the specific type of adjuvant chemotherapy administered to patients was not recorded, which means that there could have been significant differences in chemotherapy regimens between the groups—especially since the interrupted ASS group was examined before 2010 and the continuous ASS group was examined after 2010. Thirdly, our study shows data in a patient cohort taking ASS due to an underlying cardiovascular disease and not with the aim of altering the outcome of PC treatment.

## 5. Conclusions

In patients with upfront surgery for curative resection of pancreatic carcinoma, the perioperative intake of aspirin had no significant influence on overall and disease-free survival. However, randomized controlled trials are needed to clarify the influence of ASS on the long-term survival in patients with pancreatic carcinoma.

## Figures and Tables

**Table 1:** Characteristics of patients undergoing pancreatic resection for pancreatic ductal adenocarcinoma stratified to ASS intake (No ASS vs. Interrupted ASS (iASS) vs. Continuous ASS (cASS)).

	No ASS	Interrupted ASS (iASS)	Continuous ASS (cASS)	*p* (No ASS vs. ASS)	*p* (iASS vs. cASS)	*p* (No ASS vs. iASS vs. cASS)
Number	170	24	19			
Age (years), median (IQR)	67 (15)	70 (12)	74 (12)	**0.013**	0.455	**0.039**
Gender, n (%)				0.003	0.170	**0.003**
Female	88 (52)	4 (17)	7 (37)			
Male	82 (48)	20 (83)	12 (63)			
ASA, n (%)				0.064	0.173	0.079
I	3 (2)	1 (4)	0 (0)			
II	107 (63)	12 (50)	6 (32)			
III	50 (29)	11 (46)	11 (58)			
Unknown	10 (6)	0 (0)	2 (11)			
BMI (kg/m2), median (IQR)	25.6 (4.8)	25.0 (4.7)	26.7 (5.9)	0.858	0.664	0.886
Alcohol abuse (n = 187) *, n (%)	85 (57)	11 (46)	6 (40)	0.149	0.753	0.301
Nicotine abuse (n = 208) *, n (%)	39 (24)	6 (25)	2 (11)	0.545	0.270	0.450
Comorbidity, n (%)						
Hypertension	82 (48)	21 (88)	15 (79)	<0.001	0.680	<0.001
Diabetes	41 (24)	10 (42)	7 (37)	0.055	0.765	0.126
Cardiovascular disease	10 (6)	8 (33)	8 (42)	<0.001	0.752	<0.001
Pulmonary disease	13 (8)	6 (25)	0 (0)	0.229	0.027	0.009
Cerebrovascular disease	6 (4)	4 (17)	2 (11)	0.017	0.678	0.025
Liver disease	13 (8)	1 (4)	2 (11)	1.000	0.575	0.811
Preoperative biliary stenting, n (%)	87 (51)	13 (54)	12 (63)	0.490	0.530	0.561
Preoperative hemoglobin (g/dL), median (IQR)	12.9 (2.3)	13.1 (1.6)	12.6 (2.5)	0.754	0.533	0.789
Preoperative WBC (109/L), median (IQR)	7.0 (3.7)	6.9 (2.3)	6.9 (3.6)	0.873	0.772	0.962
Preoperative platelets (103/uL), median (IQR)	261 (128)	247 (100)	243 (107)	0.244	0.816	0.482
Preoperative albumin (g/L), median (IQR)	40.3 (6.6)	41.1 (9.1)	38.6 (7.6)	0.757	0.395	0.610
Preoperative CRP (mg/L), median (IQR)	6 (19)	5 (11)	6 (7)	0.676	0.703	0.915
Preoperative CA19-9 (U/mL) (n = 191) *, median (IQR)	92 (404)	105 (290)	64 (90)	0.951	0.354	0.701
Preoperative CEA (ng/mL) (n = 155) *, median (IQR)	2.4 (3.1)	2.8 (3.3)	1.8 (3.4)	0.587	0.216	0.450

ASA = American Society of Anesthesiologists classification; BMI = body mass index; WBC = white blood cells; CRP = C-reactive protein. * Missing data.

**Table 2:** Surgical and histopathological details of patients undergoing pancreatic resection for pancreatic ductal adenocarcinoma stratified to ASS intake (No ASS vs. Interrupted ASS (iASS) vs. Continuous ASS (cASS)).

	No ASS (n = 170)	Interrupted ASS (iASS) (n = 24)	Continuous ASS (cASS) (n = 19)	*p* (No ASS vs. ASS)	*p* (iASS vs. cASS)	*p* (No ASS vs. iASS vs. cASS)
Kind of surgery				0.393	1.000	0.724
Pancreatic head resection	127 (75)	20 (83)	15 (79)			
Distal pancreatectomy	36 (21)	4 (17)	4 (21)			
Total pancreatectomy	7 (4)	0 (0)	0 (0)			
Vascular resection, n (%)	42 (25)	10 (42)	7 (37)	0.059	0.765	0.149
Multivisceral resection, n (%)	29 (17)	3 (13)	6 (32)	0.656	0.153	0.217
Operative time (min), median (IQR)	278 (104)	284 (97)	293 (114)	0.174	0.236	0.245
Intraoperative blood loss (ml), median (IQR)	550 (668)	500 (950)	750 (650)	0.647	0.932	0.898
Intraoperative blood transfusion, n (%)	44 (26)	7 (29)	8 (42)	0.256	0.521	0.357
T category				0.859	0.136	0.465
pT1	10 (6)	1 (4)	1 (5)			
pT2	29 (17)	2 (8)	6 (32)			
pT3	127 (75)	21 (88)	12 (63)			
pT4	4 (2)	0 (0)	0 (0)			
N category				0.729	1.000	0.903
pN0	70 (41)	9 (38)	7 (37)			
pN+	100 (59)	15 (62)	12 (63)			
M category				0.379	0.678	0.422
M0	156 (92)	20 (83)	17 (90)			
M1	14 (8)	4 (17)	2 (10)			
R status				0.111	1.000	0.348
R0	152 (89)	19 (79)	15 (79)			
R1	14 (8)	3 (13)	3 (16)			
R2	4 (2)	2 (8)	1 (5)			
Differentiation				0.067	0.256	0.080
G1	2 (1)	2 (8)	1 (5)			
G2	59 (35)	9 (38)	3 (16)			
G3	109 (64)	13 (54)	15 (79)			

**Table 3:** Outcome parameters of patients undergoing pancreatic resection for pancreatic ductal adenocarcinoma stratified to ASS intake (No ASS vs. Interrupted ASS (iASS) vs. Continuous ASS (cASS)).

	No ASS (n = 170)	Interrupted ASS (iASS) (n = 24)	Continuous ASS (cASS) (n = 19)	*p* (No ASS vs. ASS)	*p* (iASS vs. cASS)	*p* (No ASS vs. iASS vs. cASS)
Morbidity, n (%)	109 (64)	13 (54)	10 (53)	0.221	1.000	0.448
Major morbidity, n (%)	48 (28)	8 (33)	4 (21)	1.000	0.500	0.697
Mortality, n (%)	5 (3)	1 (4)	2 (10)	0.363	0.575	0.198
Re-operation, n (%)	17 (10)	2 (8)	0 (0)	0.376	0.495	0.348
POPF, n (%)	37 (22)	3 (13)	1 (5)	0.082	0.618	0.160
DGE, n (%)	54 (32)	7 (29)	4 (21)	0.465	0.728	0.630
PPH, n (%)	1 (1)	0 (0)	0 (0)	1.000	-	1.000
Length of postoperative stay (days), median (IQR)	18 (13)	16 (11)	16 (13)	0.183	0.304	0.220
Adjuvant chemotherapy, n (%)	91 (54)	14 (58)	10 (53)	0.865	0.764	0.907
Overall survival (months), median (SD)	21.6 (2.7)	17.8 (1.8)	19.6 (10.0)	0.309	0.567	0.489
Disease-free survival * (months), median (SD)	14.7 (1.8)	14.0 (4.5)	18.3 (2.8)	0.797	0.906	0.957

POPF = postoperative pancreatic fistula; DGE = delayed gastric emptying; PPH = post-pancreatectomy hemorrhage; SD = standard deviation. * only patients after R0 resection > n = 186.

**Table 4:** Prognostic factors of patients with resected pancreatic ductal adenocarcinoma for overall survival (OS).

	Overall Survival (OS)							
		Univariate				Multivariate		
	n	Median OS	2-YSR (%)	5-YSR (%)	*p*	HR	95% CI	*p*
Age					**<0.001**			
≤70 years	123	29.2	56.0	28.2		1.0		
>70 years	90	17.1	32.7	16.1		2.2	1.6–3.1	**<0.001**
Gender					0.119			
Female	99	26.8	53.4	27.8				
Male	114	19.8	39.3	18.3				
ASA (n = 201) *					**0.020**			
I/II	129	24.1	51.0	27.0		1.0		
III	72	16.9	38.5	15.2		1.4	1.0–1.9	0.088
Ca19-9 (n = 191) *					0.173			
<50 U/ml	72	24.2	51.9	30.8				
≥50 U/ml	119	20.7	45.1	19.5				
ASS intake					0.489			
No ASS	170	21.6	47.2	24.3		1.0		
iASS	24	17.8	41.7	22.2		0.9	0.5–1.5	0.704
cASS	19	19.6	40.5	13.5		1.1	0.6–2.1	0.681
Kind of surgery					0.153			
Pancreatic head resection	162	23.4	49.5	23.2				
Distal pancreatectomy	44	17.3	38.6	22.7				
Total pancreatectomy	7	6.7	14.3	14.3				
Vascular resection					0.242			
Yes	59	17.2	44.3	22.3				
No	154	22.0	46.8	23.5				
Multivisceral resection					0.111			
Yes	38	17.0	31.6	18.9				
No	175	23.4	49.2	23.8				
T category					**0.013**			
pT1/pT2	49	37.8	65.0	33.2		1.0		
pT3/pT4	164	18.4	40.7	20.3		1.3	0.8–2.1	0.243
N category					**<0.001**			
pN0	86	39.7	60.4	35.9		1.0		
pN+	127	17.8	36.5	14.8		2.0	1.4–2.9	**<0.001**
M category					**0.010**			
M0	193	23.1	48.8	24.5		1.0		
M1	20	12.4	20.0	10.0		0.8	0.4–1.6	0.616
R status					**<0.001**			
R0	186	23.8	49.6	25.6		1.0		
R1/R2	27	8.8	21.2	5.3		2.8	1.6–4.8	**<0.001**
Differentiation					**<0.001**			
G1/G2	76	37.8	60.4	34.4		1.0		
G3	137	17.1	37.9	16.2		1.7	1.2–2.5	**0.005**
Morbidity					0.376			
Yes	132	19.8	41.6	22.9				
No	81	29.2	53.7	22.0				
Re-operation					0.131			
Yes	19	17.0	36.8	12.3				
No	192	22.0	47.5	24.4				
Adjuvant chemotherapy					0.297			
Yes	115	23.4	48.5	22.5				
No	98	17.2	42.9	23.0				

ASA = American Society of Anesthesiologists classification; * missing data.

**Table 5:** Prognostic factors of patients with resected pancreatic ductal adenocarcinoma for disease-free survival (DFS).

	Disease-Free Survival (DFS) **							
		Univariate				Multivariate		
	n	Median OS	2-YSR (%)	5-YSR (%)	*p*	HR	95% CI	*p*
Age					**0.044**			
≤70 years	107	16.8	40.3	26.6		1.0		
>70 years	79	11.1	28.4	16.2		1.5	1.0–2.1	**0.040**
Gender					0.164			
Female	88	16.2	41.8	26.6				
Male	98	14.0	29.3	17.7				
ASA (n = 177) *					0.166			
I/II	118	14.9	33.9	26.5				
III	59	14.0	35.6	11.6				
Ca19-9 (n = 168) *					0.077			
<50 U/ml	66	18.4	42.9	27.9				
≥50 U/ml	102	12.6	32.6	19.5				
ASS intake					0.957			
No ASS	152	14.7	35.2	23.3		1.0		
iASS	19	14.0	36.8	15.8		0.9	0.5–1.6	0.829
cASS	15	18.3	33.3	16.7		1.1	0.6–2.1	0.778
Kind of surgery					0.398			
Pancreatic head resection	145	16.2	39.1	21.4				
Distal pancreatectomy	38	9.7	21.1	21.1				
Total pancreatectomy	3	15.2	33.3	33.3				
Vascular resection					0.626			
Yes	47	12.1	34.1	25.6				
No	139	14.9	35.6	21.7				
Multivisceral resection					0.235			
Yes	28	9.0	25.0	21.4				
No	158	14.9	37.1	22.4				
T category					**0.009**			
pT1/pT2	45	22.8	48.0	34.4		1.0		0.141
pT3/pT4	141	12.6	31.2	18.5		1.4	0.9–2.2	
N category					**<0.001**			
pN0	76	20.0	48.5	34.8		1.0		
pN+	110	12.1	26.3	13.6		1.8	1.2–2.6	**0.002**
M category					**0.005**			
M0	173	16.0	37.3	23.1		1.0		
M1	13	7.8	7.7	7.7		1.6	0.9–3.1	0.126
Differentiation					**0.008**			
G1/G2	69	19.7	44.9	30.7		1.0		
G3	117	12.6	29.4	16.5		1.5	1.0–2.1	**0.037**
Morbidity					0.885			
Yes	113	14.0	37.1	22.4				
No	73	16.4	32.0	21.9				
Re-operation					0.472			
Yes	15	14.0	33.3	16.7				
No	169	14.8	35.8	22.8				
Adjuvant chemotherapy					0.790			
Yes	98	16.0	32.9	21.6				
No	88	12.6	37.5	23.0				

ASA = American Society of Anesthesiologists classification; * missing data; ** only patients after R0 resection > n = 186.

## Data Availability

The data presented in this study are available upon reasonable request from the corresponding author.
